# EGFRvIII does not affect radiosensitivity with or without gefitinib treatment in glioblastoma cells

**DOI:** 10.18632/oncotarget.5293

**Published:** 2015-09-16

**Authors:** Nina Struve, Matthias Riedel, Alexander Schulte, Thorsten Rieckmann, Tobias J. Grob, Andreas Gal, Kai Rothkamm, Katrin Lamszus, Cordula Petersen, Ekkehard Dikomey, Malte Kriegs

**Affiliations:** ^1^ Department of Radiotherapy and Radio-Oncology, University Medical Center Hamburg Eppendorf, Hamburg, Germany; ^2^ Department of Neurosurgery, University Medical Center Hamburg Eppendorf, Hamburg, Germany; ^3^ Department of Otorhinolaryngology and Head and Neck Surgery, University Medical Center Hamburg Eppendorf, Hamburg, Germany; ^4^ Department of Pathology, University Medical Center Hamburg Eppendorf, Hamburg, Germany; ^5^ Department of Human Genetics, University Medical Center Hamburg Eppendorf, Hamburg, Germany

**Keywords:** EGFRvIII, glioblastoma, radiosensitivity, X-irradiation, gefitinib

## Abstract

**Background:**

Glioblastomas (GBM) are often characterized by an elevated expression of the epidermal growth factor receptor variant III (EGFRvIII). We used GBM cell lines with native EGFRvIII expression to determine whether this EGFR variant affects radiosensitivity with or without EGFR targeting.

**Methods:**

Experiments were performed with GBM cell lines lacking (LN229, U87MG, U251, CAS-1) or endogenously expressing EGFRvIII (BS153, DKMG). The two latter cell lines were also used to establish sublines with a low (−) or a high proportion (+) of cells expressing EGFRvIII. EGFR signaling and the cell cycle were analyzed using Western blot and flow cytometry; cell survival was assessed by colony forming assay and double-strand break repair capacity by immunofluorescence.

**Results:**

DKMG and BS153 parental cells with heterogeneous EGFRvIII expression were clearly more radiosensitive compared to other GBM cell lines without EGFRvIII expression. However, no significant difference was observed in cell proliferation, clonogenicity or radiosensitivity between the EGFRvIII− and + sublines derived from DKMG and BS153 parental cells. Expression of EGFRvIII was associated with decreased DSB repair capacity for BS153 but not for DKMG cells. The effects of EGFR targeting by gefitinib alone or in combination with irradiation were also found not to depend on EGFRvIII expression. Gefitinib was only observed to influence the proliferation of EGFRvIII− BS153 cells.

**Conclusion:**

The data indicate that EGFRvIII does not alter radiosensitivity with or without anti-EGFR treatment.

## INTRODUCTION

Glioblastoma multiforme (GBM) is the most common malignant brain tumor in adult patients, with an estimated 5-year survival rate of less than 10% [1]. The current standard of care is an intensive multimodal treatment including neurosurgical resection, radiotherapy (RT) and adjuvant chemotherapy (CT) with temozolomide [2].

GBMs are generally characterized by genomic rearrangements and a variety of mutations associated with radio- and chemoresistance [3]. The most frequent alteration is the amplification of the gene encoding the epidermal growth factor receptor (EGFR), causing a massive overexpression of EGFR. This gene amplification is present in about 40%-60% of GBMs and is often associated with the expression of the deletion variant EGFRvIII. This variant lacks the exons 2–7, leading to a ligand-independent and constitutively activated receptor [4]. In GBMs, the amplified *egfr* gene is encoded on double minute chromosomes (DMC), with up to 200 copies present per nucleus [5].

There are already several pre-clinical studies analyzing the function of EGFRvIII in GBM. Due to the lack of GBM cell lines stably expressing endogenous EGFRvIII, these experiments were performed with cell lines transfected with EGFRvIII encoding vectors [6, 7]. In these studies, EGFRvIII expression was found to result not only in accelerated tumor growth but also in increased repair of X-irradiation induced DNA double-strand breaks (DSB) associated with enhanced radioresistance [6, 7]. In line with these data, the inhibition of EGFR results in a depressed DSB repair, leading in turn to radiosensitisation [6, 7].

In contrast, clinical studies investigating the potential use of EGFRvIII expression as a prognostic marker have thus far failed to yield a clear result. While small studies observed both better and poorer survival for patients with EGFRvIII positive tumors [8, 9], larger studies failed to show any association [10–13]. Likewise, no clear clinical benefit has been observed following EGFR targeting; a substantial increase in side effects was observed for this treatment, however, especially when combined with radiotherapy [14].

In this study, we analyzed the impact of EGFRvIII on cellular radiosensitivity and EGFR targeting using two GBM cell lines (DKMG and BS153) with endogenous EGFRvIII expression [5, 15]. These cell lines were also used to establish two pairs of sublines with either a low (−) or high (+) fraction of EGFRvIII expressing cells. When compared to GBM cell lines negative for EGFRvIII, DKMG and BS153 cells were found to be clearly more radiosensitive. However, using the pairs of EGFRvIII- and + sublines, we were able to demonstrate that EGFRvIII itself has no impact on either cell growth or cellular radiosensitivity with or without EGFR targeting.

## RESULTS

### Radiosensitivity of GBM cell lines with and without EGFRvIII expression

**Figure 1:**
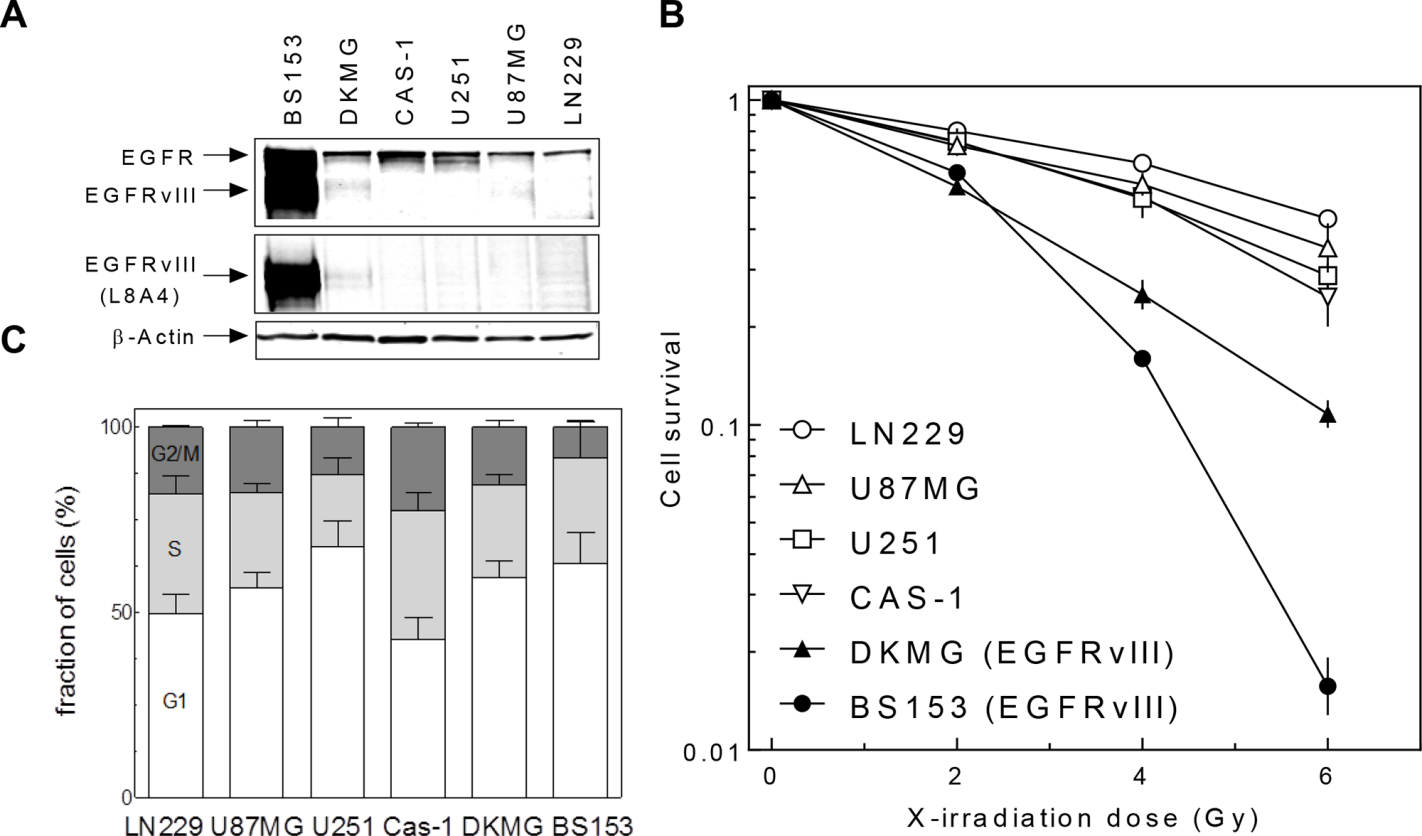
EGFRvIII expression and radiosensitivity of different GBM cell lines **A.** Expression of EGFR wildtype and EGFRvIII in different EGFRvIII positive and negative GBM cell lines as detected by Western blot using EGFR and EGFRvIII (L8A4) specific antibodies. Actin served as a loading control. **B.** Cell survival of EGFRvIII positive and negative GBM cell lines after irradiation as assesed by colony forming assay (pre-plating). **C.** Cell cycle distribution of EGFRvIII positive and negative GBM cell lines analyzed by PI staining and flow cytometry.

The radiosensitivity of six well established GBM cell lines differing in EGFRvIII expression was analyzed under normal serum conditions by colony forming assay, specifically four strains (LN229, U87MG, U251, CAS-1) without, one cell line with moderate (DKMG) and one with strong (BS153) EGFRvIII expression (Figure 1A). A pronounced difference in radiosensitivity was found for both DKMG and BS153 cells, which were clearly more sensitive, compared to GBM cell lines expressing no EGFRvIII (Figure 1B). With respect to cell cycle distribution, no obvious differences were observed between the six cell lines (Figure 1C).

### Characterization of EGFRvIII− and + sublines

**Figure 2:**
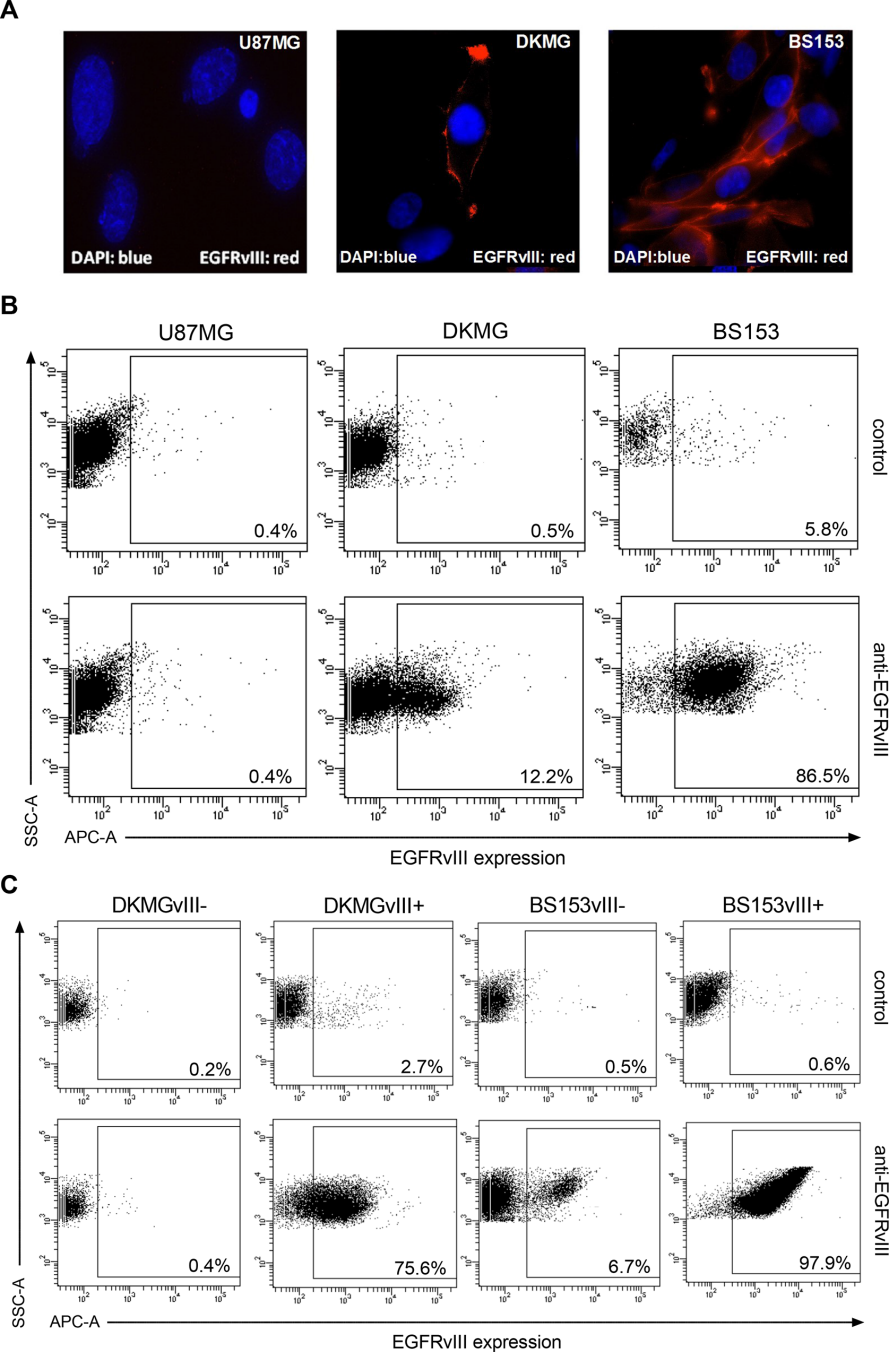
Generation of EGFRvIII− and EGFRvIII+ sublines as determined via FACS **A.** Detection of EGFRvIII (red) by immunofluorescence using an EGFRvIII-specific antibody (blue, DNA staining with DAPI). **B.** EGFRvIII expression in parental DKMG and BS153 cells as detected by flow cytometry (dot plots). EGFRvIII expression (APC-A) was detected using an EGFRvIII-specific antibody. U87MG cells served as a negative control (SSC-A; side scatter) and a secondary antibody control was used to asses unspecific staining. **C.** Using parental DKMG and BS153 cell lines, EGFRvIII expressing and non-expressing cells were seperated by FACS to generate EGFRvIII− and EGFRvIII+ sublines. EGFRvIII expression in EGFRvIII−/+ sublines 4-6 passages after sorting as analyzed by flow cytometry.

Immunofluorescent staining of EGFRvIII revealed that its expression is heterogeneous in DKMG as well as BS153 cells, with mostly membranous localization (Figure 2A). The detection of EGFRvIII by flow cytometry showed a great difference in the fraction of cells positive for EGFRvIII, with only 11.7% for DKMG and 80.7% for the BS153 culture. In addition, the expression was clearly higher for BS153 cells (Figure 2B).

To establish EGFRvIII negative (−) and positive (+) sublines from DKMG and BS153 cell lines, the EGFRvIII was marked by a specific antibody and EGFRvIII− and + cells were separated by fluorescence activated cell sorting (FACS). The EGFRvIII−/+ subclones were grown in 10% heat inactivated FCS and four to six passages after sorting, the fraction of cells expressing EGFRvIII was found to be 72.9% (DKMG) and 97.3% (BS153) in the EGFRvIII+ cultures, but only 0.2% (DKMG) and 6.2% (BS153) in the EGFRvIII- cultures (Figure 2C). These percentages remained constant for up to 20 passages or 18 weeks respectively with little variation (Supplementary Figure S1). Whereas presence of the EGFRvIII gene is frequently associated with EGFR gene amplification [16], EGFRvIII protein expression is a dynamic process regulated also by epigenetic events [5, 17]. Therefore changes in the EGFRvIII expression profile—presence of EGFRvIII expressing cells in the EGFRvIII− subline and *vice versa*—are expected especially since we still detect EGFR gene amplification in EGFRvIII- DKMG and BS153 cells (data not shown).

### Impact of EGFRvIII on cell signaling and cell growth

**Figure 3:**
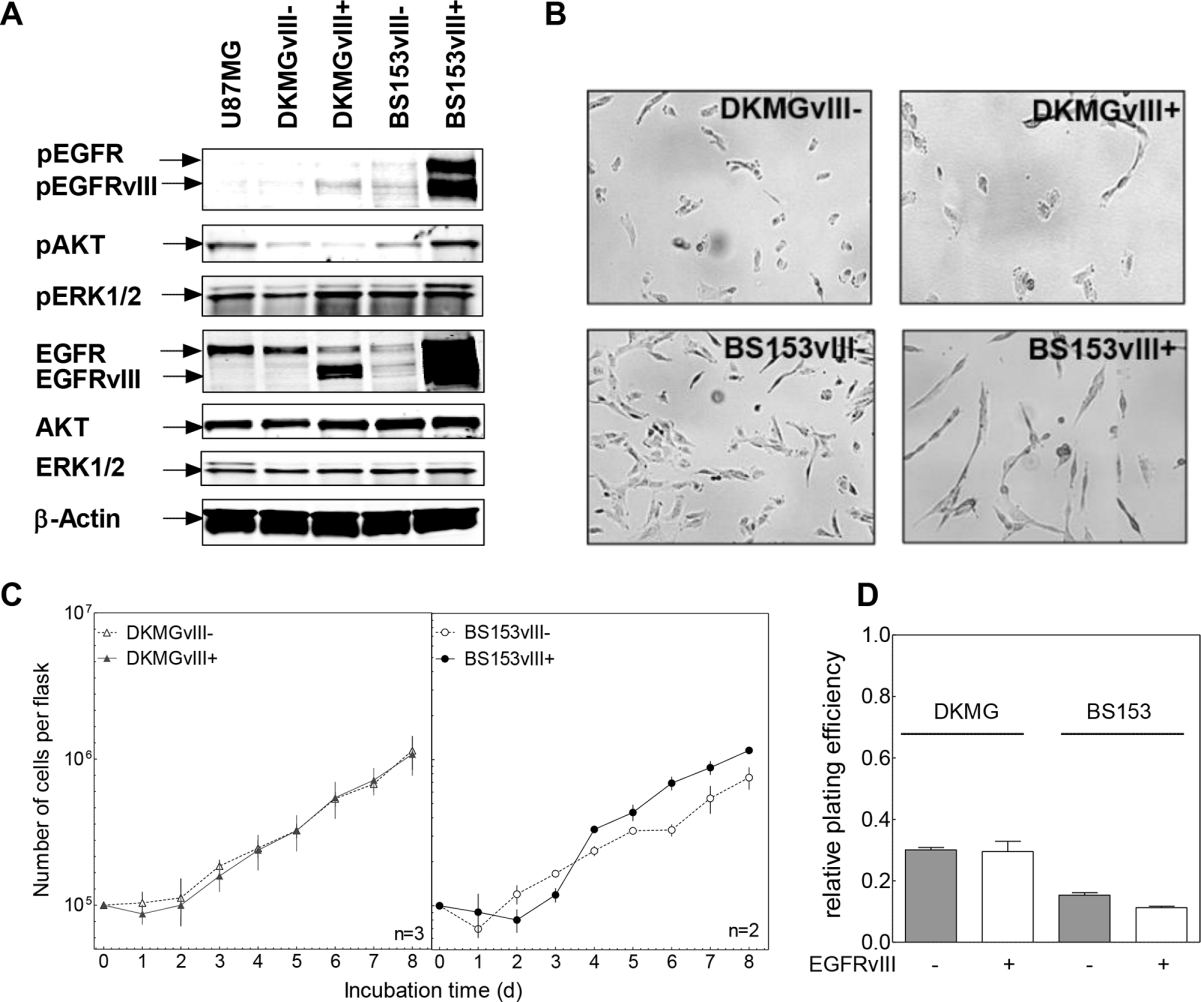
Impact of EGFRvIII expression on EGFR downstream signaling, proliferation and clonogenicity **A.** Effect of EGFRvIII expression on EGFR signaling as determined by Western blot analysis using antibodies against (p)EGFR (Y1173), (p)AKT (T308) and (p)ERK1/2 (T202/Y204). U87MG cell served as a negative control cell line. **B.** Morphology of EGFRvIII−/+ DKMG and BS153 cells (phase-contrast microscopy, 100× magnification). **C.** Proliferation of EGFRvIII− and EGFRvIII+ DKMG and BS153 cells. The cell number was determind for up to 8 days. **D.** Relative clonogenicity of EGFRvIII−/+ DKMG and BS153 cells as determined by colony forming assay (pre-plating).

To study the effect of EGFRvIII on cellular signaling, cell lysates from DKMG and BS153 EGFRvIII− and + sublines were analyzed by Western blot. EGFRvIII+ BS153 cells showed an enhanced activation/phosphorylation of EGFR, EGFRvIII, AKT and ERK1 (upper band) compared to the EGFRvIII− subline. In contrast, for EGFRvIII+ DKMG cells, an enhanced phosphorylation of EGFRvIII and ERK2 (Figure 3A, lower band) was detectable, when compared to EGFRvIII− DKMG cells, indicating that the effects of EGFRvIII expression on down-stream signaling differ in these two cell lines.

Noticeable morphological changes were seen depending on the EGFRvIII status for BS153, but not for DKMG cells (Figure 3B). EGFRvIII− cells displayed a flattened shape in contrast to the more spindle-like morphology of EGFRvIII+ cells, as has also been reported previously [5]. With respect to cell growth, no differences were observed for the DKMG sublines, and only a minor differences seen in the BS153 sublines, with EGFRvIII+ cells showing a slightly delayed proliferation in the first days after seeding (Figure 3C) and a small reduction in plating efficiency (Figure 3D).

### Impact of EGFRvIII on DNA double-strand break (DSB) repair and radiation response

**Figure 4:**
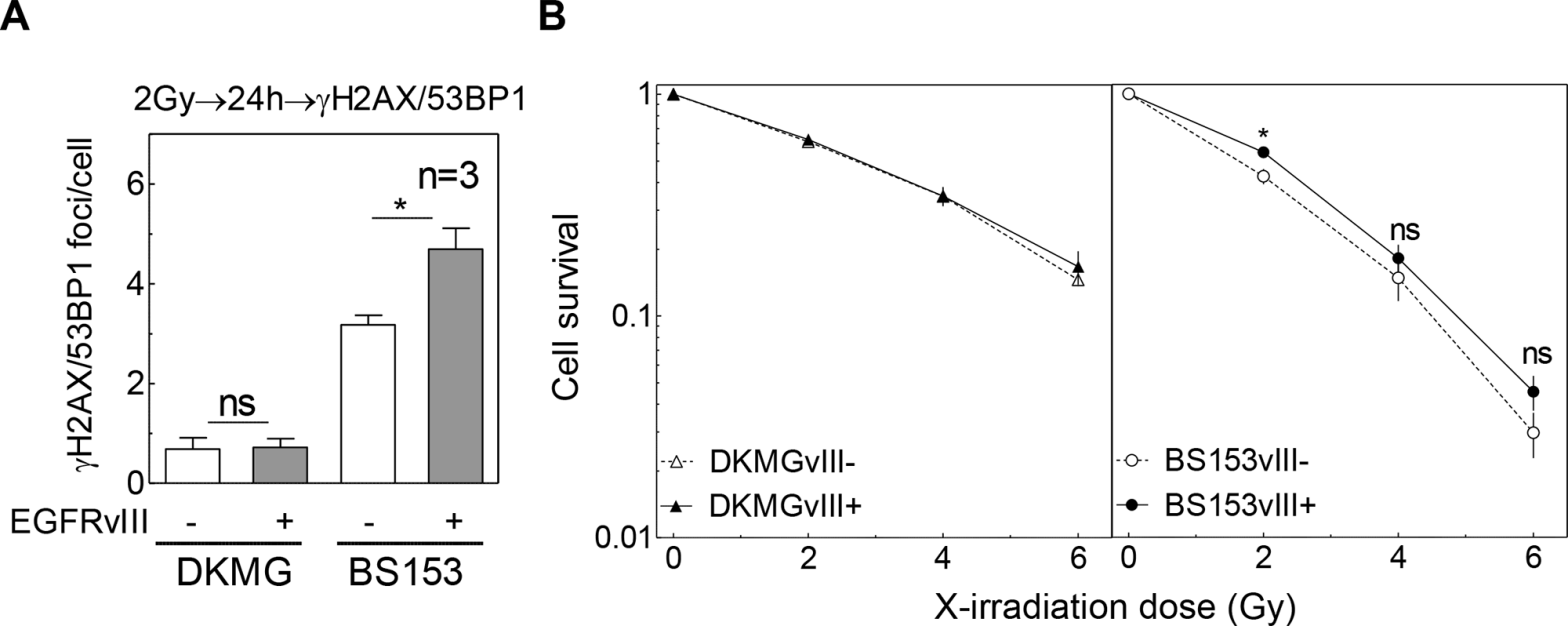
Impact of EGFRvIII expression on DSB repair capacity and cellular radiosensitivity **A.** Quantification of residual γH2AX/53BP1 double positive foci in DKMGvIII−/+ and BS153vIII−/+ cells 24 h after irradiation with 2 Gy. **B.** Cell survival after irradiation as assessed by colony forming assay (pre-plating).

Previous data using cell lines transfected with EGFRvIII encoding vectors demonstrated that DSB repair after irradiation was increased due to EGFRvIII expression, leading to a radioresistance when EGFRvIII was expressed [6]. To study the effect of endogenous EGFRvIII on DSB repair, the DKMG and BS153 sublines were exposed to 2 Gy and the number of co-localized γH2AX/53BP1 repair foci was measured 24 h after irradiation (Supplementary Figure S2, Figure 4A). No difference was observed for the two DKMG sublines, while a significant increase in the number of residual γH2AX/53BP1 foci was detected for EGFRvIII+ BS153 cells, indicating a reduction in DSB repair capacity (Figure 4A). With regard to cellular radiosensitivity, no significant difference was detectable between the EGFRvIII− and + sublines for DKMG cells. For BS153 cells, a slight increase in the surviving fraction of the EGFRvIII+ subline was observed, although this was not significant at 4 and 6 Gy (Figure 4B; p_2Gy_ = 0.03, p_4Gy_ = 0.46, p_6Gy_ = 0.22). Additionally, knock down of EGFRvIII via siRNA did not change the cellular radiosensitivity of BS153 cells (Supplementary Figure S3).

These results show that endogenously encoded EGFRvIII appears to have no or even a negative impact on DSB repair and fails to confer cellular radioresistance.

### Impact of EGFRvIII on EGFR targeting

**Figure 5:**
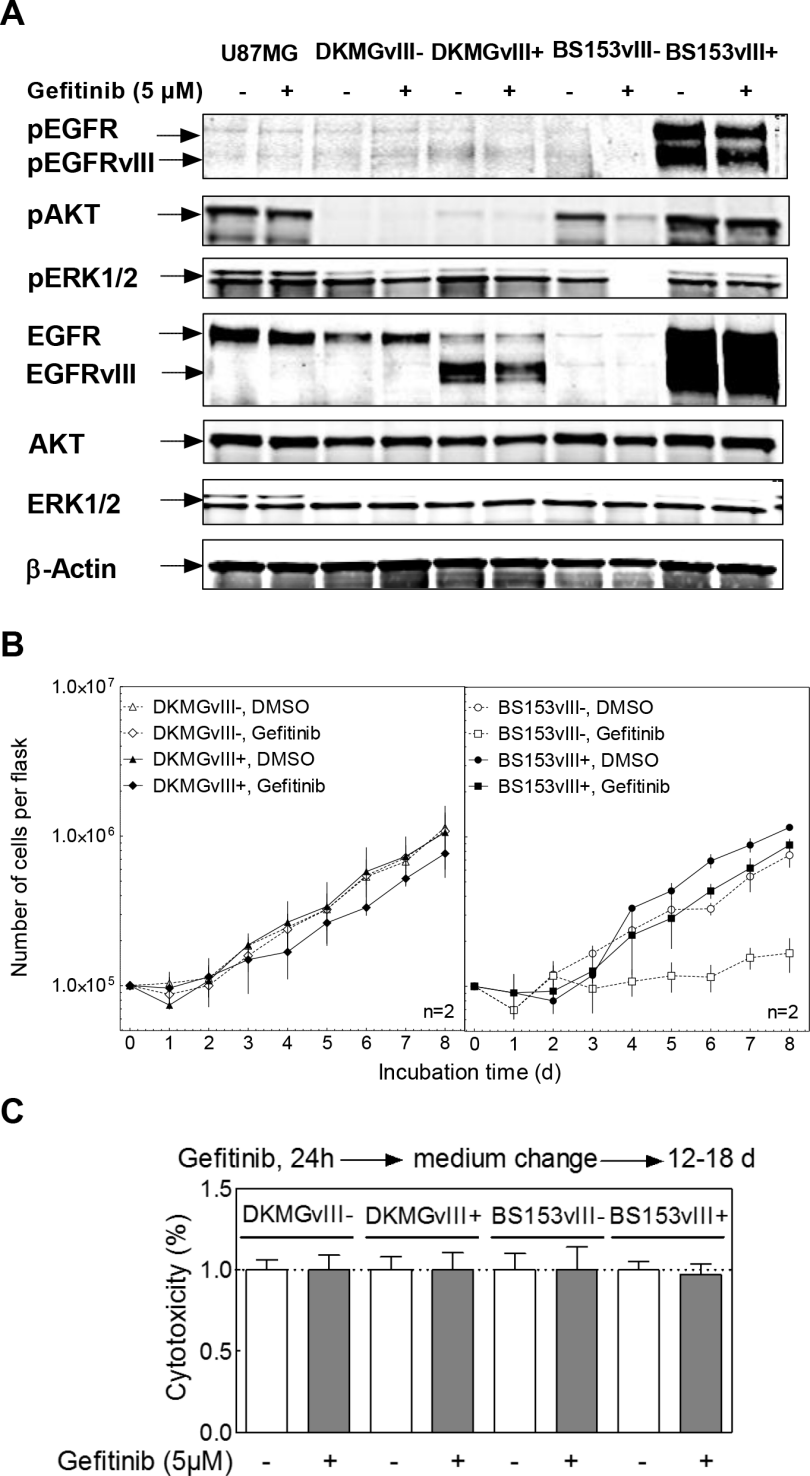
Effect of gefitinib on EGFR signaling, proliferation and clonogenicity DKMGvIII−/+ and BS153vIII−/+ cells were treated with 5 μM gefitinib. **A.** After 2 h incubation, phosphorylation of EGFR (Y1173), AKT (T308) and ERK1/2 (T202/Y204) was determined by Western blot analysis using phosphospecific antibodies. The detection of unphosphorylated proteins and actin served as controls. **B.** Proliferation of DKMGvIII−/+ and BS153vIII−/+ cells in the presence of gefitinib (*n* = 2). The cell number was determind for up to 8 days. The data set from Figure 3C was used for comparison with untreated cells. **C.** Relative cytotoxicity of gefitinib as determind by colony forming assay (pre-plating). The surviving fraction of gefitinib-treated cells was normalized to the plating efficiency of untreated cells.

We also tested whether EGFRvIII influences EGFR targeting by using the small molecule inhibitor gefitinib applied either alone or in combination with IR. For DKMG and BS153 EGFRvIII+ sublines, a minor inhibition of EGFR-signaling (phosphorylation of EGFR, EGFRvIII, AKT and ERK1/2) (Figure 5A) and proliferation (Figure 5B) was detectable, while no effect was detectable in the EGFRvIII− DKMG subline. In contrast, gefitinib was found to cause a clear inhibition of EGFR signaling (Figure 5A) associated with a strong reduction in cell proliferation (Figure 5B) in the EGFRvIII− BS153 subline. However, no cytotoxic effect was observed in any of the four sublines when the cells were treated with gefitinib for 24 h (Figure 5C).

**Figure 6:**
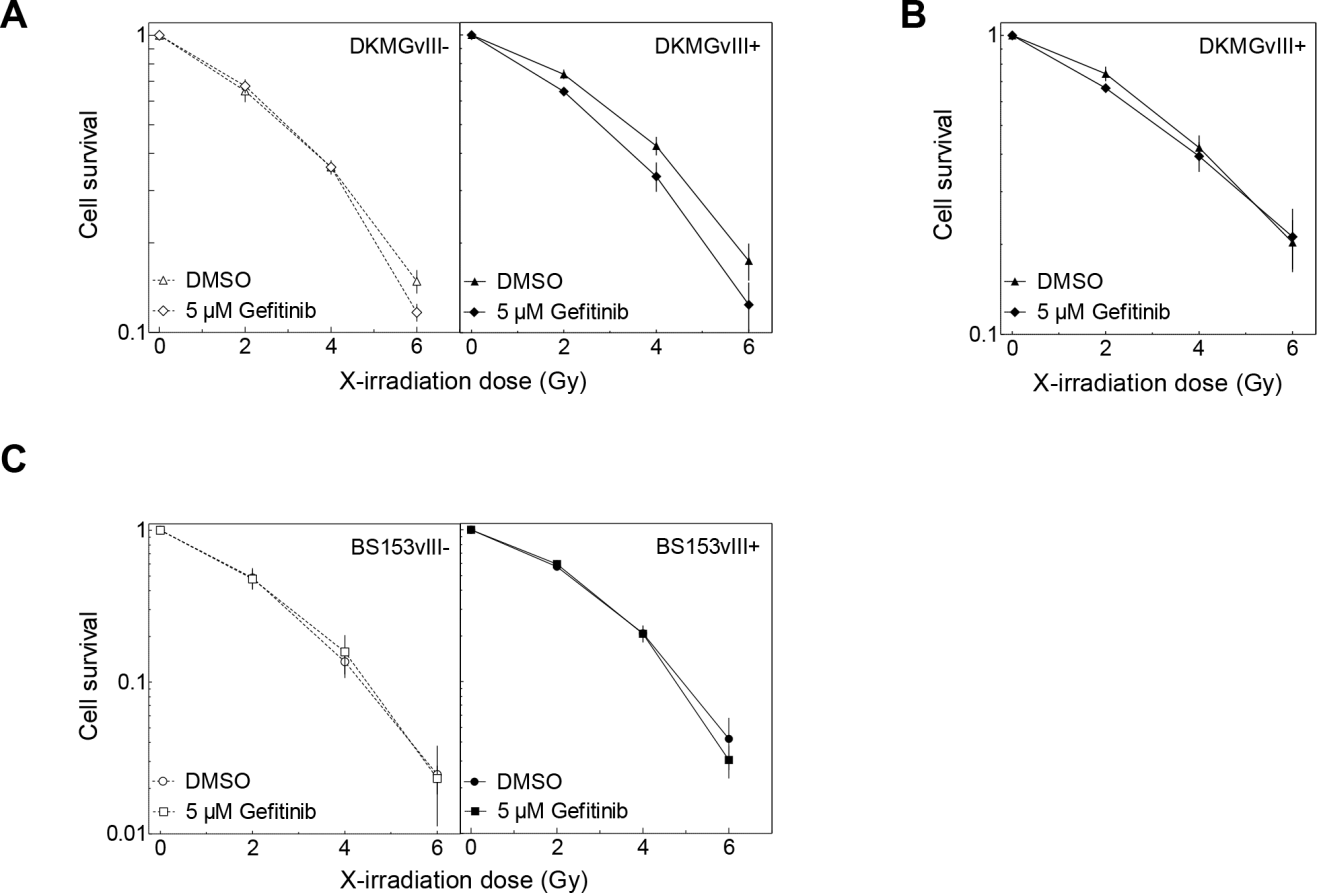
Effect of gefitinib on radiosensitivity DKMGvIII−/+ and BS153vIII−/+ cells were treated with gefitinib for 2 h before irradiation. Cell survival of DKMGvIII−/+ and BS153vIII−/+ cells was assesed by colony forming assay **A, C.** under pre-plating conditions and **B.** delayed plating conditions (only DKMGvIII+).

The effect of gefitinib on cell survival after irradiation was tested by adding gefitinib 2 h prior IR, followed by a 24 h incubation, after which gefitinib was removed and the cells were retained for colony growth (pre-plating). A change in cellular radiosensitivity induced by gefitinib was only seen for EGFRvIII+ DKMG cells (Figure 6A). However, when these cells were trypsinized and replated for colony growth 24 h after irradiation (delayed-plating), this radiosensitization was completely abolished (Figure 6B). For BS153, radiosensitivity was not affected in either EGFRvIII− or EGFRvIII+ cells (Figure 6C).

## DISCUSSION

The aim of this study was to determine the impact of EGFRvIII on the sensitivity of GBM cells towards IR and anti-EGFR targeting. So far, such studies have been performed with cell lines transfected with EGFRvIII expressing vectors [6, 7, 18]. The analysis in the present study was performed for the first time with GBM cell lines expressing endogenous EGFRvIII, with DKMG cells showing more than 10% and BS153 more than 80% of cells positive for EGFRvIII.

Both DKMG and BS153 cells were used to establish sublines with either a very low (EGFRvIII− ) or very high fraction of EGFRvIII positive cells (EGFRvIII+). These sublines were found to differ in EGFR expression: The BS153 EGFRvIII+ subline is characterized by a strong expression of EGFR and EGFRvIII, while the DKMG EGFRvIII+ subline showed only a low expression of wild type EGFR and a moderate expression of EGFRvIII. This divergence in EGFR and EGFRvIII expression might explain the differences observed concerning downstream signaling, proliferation and morphology. For DKMG cells, the EGFRvIII+ subline exhibited merely greater ERK2 phosphorylation, while for BS153 cells, the EGFRvIII+ subline displayed both greater ERK1 and AKT phosphorylation, which is in line with former siRNA data analyzing erlotinib resistant BS153 with elevated EGFRvIII level [19]. Additionally a slightly impaired cell growth and an altered cell morphology can be observed for the BS153 EGFRvIII+ subline when compared to the EGFRvIII- subline. The only difference observed in terms of DSB repair was also seen in the BS153 cells, with the EGFRvIII+ subline showing an impaired repair capacity. In this context p53 might be of importance, since BS153 sublines are mutated in p53 (R248Q) while DKMG sublines—which did not display any differences in DSB repair—are wild type (see *Materials and Methods*).

Strikingly, no significant difference in cellular radiosensitivity was observed for either of the two sublines derived from DKMG or BS153 cells. This observation suggests that EGFRvIII has no impact on the cellular radiosensitivity in these GBM cell lines. Surprisingly, when compared to GBM cell lines lacking EGFRvIII expression (LN229, U87MG, U251, CAS-1), both parental EGFRvIII positive cell lines showed a higher radiosensitivity (Figure 1B). Since this difference cannot be attributed to the presence of EGFRvIII, other still unknown factors appear to play a role, with genetic differences between EGFR positive and negative GBM cell lines likely of importance. However, a larger number of EGFRvIII positive and negative GBM cell lines still needs to be analyzed in order to bring clarity to this issue.

Using the DKMG and BS153 sublines, we also tested whether EGFRvIII may influence the cell's response to EGFR targeting. In order to inhibit EGFR and EGFRvIII signaling we used gefitinib, a small molecule inhibitor used in clinical trials for the treatment of GBM patients [20, 21]. Again, greater effects were observed for the BS153 sublines, which showed a strong inhibition of EGFR downstream signaling and a suppressed proliferation in EGFRvIII−, but not in EGFRvIII+ cells. Nevertheless, no notable cytotoxic effect was detected in any of the four EGFRvIII sublines after treatment with gefitinib alone or after combined treatment with irradiation. Even the small increase in radiosensitivity seen for the EGFRvIII+ DKMG subline treated by gefitinib was found to be abolished when cells were replated 24 h after irradiation. This is assumed to result from a reversible cell cycle arrest induced in p53wt DKMG cells through combined treatment with EGFR inhibition and irradiation, as recently demonstrated for p53wt NSCLC cells [22]. Overall, these data demonstrate that both the cytotoxic as well as the cytostatic effect of EGFR targeting in GBM cells does not depend on EGFRvIII.

In contrast to the results presented here, strong effects have been reported for EGFRvIII negative cell lines transfected with EGFRvIII expressing vectors. EGFRvIII was found not only to strongly enhance DSB repair and to increase radioresistance, but also to accelerate tumor growth [6, 7, 18]. In line with this, a reduction in DSB repair and radioresistance was observed when EGFRvIII transfected cells were treated with EGFR inhibitors. Taken together, these data show that the effects of EGFRvIII in transfected cells are different from those resulting from endogenous expression and highlight the importance of the experimental model system. In this context our established isogenetic sublines facilitate the analysis of EGFRvIII-mediated effects without cell engineering.

Our data suggest that the level of EGFRvIII expression in GBM tumors cannot serve as a predictive marker for increased radioresistance. This conclusion is in line with recent clinical data showing a lack of association between EGFRvIII expression and progression free and overall survival for GBM patients treated with surgery, RT and CT according to current standards [13]. Recent clinical trials have also failed to show a benefit of EGFR targeting for the outcome of patients with GBM tumors, while simultaneously demonstrating an increase in normal tissue damage [14, 23, 24]. Our data further indicate that EGFR targeting alone or in combination with RT does not contribute to enhanced control of GBM tumors, even when these are positive for EGFRvIII expression.

In summary, it is shown here for the first time that endogenous EGFRvIII in GBM cells does not affect radiosensitivity with or without EGFR targeting. Nevertheless, the isogenetic EGFRvIII− and EGFRvIII+ sublines established here can be considered to be an optimal tool for the analysis of the impact of EGFRvIII on other parameters relevant for the outcome of GBM tumor patients.

## MATERIALS AND METHODS

### Inhibitors and reagents

To inhibit EGFR and EGFRvIII activity, 5 μM gefitinib (LC Laboratories, tyrosine kinase inhibitor) dissolved in DMSO (Sigma) was used.

### Cell culture

BS153 cells were generated by Jones et al. [15]. DKMG cell were obtained from the DSMZ (Germany) and Cas-1 cells were obtained from Banca Biologica e Cell Factory (Italy). BS153, LN229, U87MG, U251 and Cas-1 cells were cultured in DMEM (Sigma-Aldrich) supplemented with 10% FCS (Biochrome), 2 mM L-glutamine and 1 mM sodium pyruvate (Sigma-Aldrich); DKMG cells were cultured in RPMI (10%FCS, 2 mM L-glutamine, 1 mM sodium pyruvate). In accordance to Schulte et al. [25] all sublines derived from BS153 and DKMG cells were grown in 10% heat inactivated FCS to maintain EGFRvIII expression in the EGFRvIII+ sublines. All cells were cultured at 37°C, 5% CO_2_ and 100% humidification. Sequencing of the PTEN gene (exons 1–9) revealed mutations in DKMG (T167A) and BS153 (C136Y) cells (Table S1). Sequencing of the TP53 gene (exons 5–8) confirmed a wild type sequence in DKMG and revealed a R248Q mutation in BS153 cells (Supplementary Table S1). All cells were identified by a short tandem repeat multiplex assay (Applied Biosystems; Supplementary Table S2). The morphology of cells was recorded using a phase-contrast microscopy with 100x magnification (Zeiss Axioplan 2).

### Irradiation

Cells were irradiated at room temperature with 200 kV X-rays (Gulmay RS225, Gulmay Medical Ltd., 15 mA, 0.8 mm Be + 0.5 mm Cu filtering) at a dose rate of 1.2 Gy/min.

### Cell survival

Cell survival was determined using the colony forming assay. For pre-plating, 250–350 cells were seeded per 6-well plate 24 h prior to treatment. When incubated with gefitinib, the medium was replaced 24 h after treatment, followed by further incubation with AmnioMax C-100 Basal Medium (Life Technologies) containing 10% FCS and C-100 supplement (Life Technologies) to optimize colony formation. For delayed plating experiments, 1×10^5^ cells were seeded per flask and grown for 6 days to achieve an exponentially growing culture before treatment. Twenty-four hours after treatment, cells were re-seeded for colony formation under the conditions described above. Colonies were allowed to grow for 2 to 3 weeks depending on the treatment in order to adapt for growth delay. Colonies were then fixed in 70% ethanol and stained with crystal violet; colonies of more than 50 cells were counted. The surviving fraction of irradiated or gefitinib-treated cells was normalized to the plating efficiency of untreated cells.

### Cell cycle analysis

For cell cycle analysis, cells were harvested, fixed by 70% ethanol and stored at −20°C. Thereafter cells were washed with PBS (0.1% Tween) and the DNA was stained with propidium iodide (PI, 10 μg/ml) containing RNase A (RNase A 0.1 μg/ml) for 30 min at room temperature. DNA histograms were constructed using flow cytometry (FACS Scan Canto and FACSDiva software, BD Biosciences) and the fraction of G1, S and G2 phase cells was calculated using ModFit LT™ software (Verity Software House, Inc.).

### Western blot

Proteins from whole cell extracts were detected by Western blot according to standard protocols. The antibodies recognizing EGFR, pEGFR (Y1173), ERK, pERK (T202, Y204), AKT and pAKT (T308) were obtained from Cell Signaling Technology, while the anti β-Actin antibody was purchased from Sigma-Aldrich. The EGFRvIII antibody (clone L8A4) was kindly provided by D. Binger. Secondary anti-mouse and anti-rabbit antibodies were purchased from LI-COR Biosciences. The Odyssey^®^ CLx Infrared Imaging System (LI-COR Biosciences) was used for signal detection and quantification.

### Immunofluorescence

Immunofluorescent staining was performed as described earlier [26]. For the detection of EGFRvIII, cells were fixed (4% formalin, 15 min, RT) and stained with a primary antibody (L8A4). For detection of γH2AX/53BP1 co-localized DSB repair foci, cells were fixed and incubated with anti-phospho-Histone H2AX (Ser139, Upstade) and anti-53BP1 (Novus, Biologicals) antibodies with the respective secondary antibodies (fluorescein-labeled anti-rabbit antibody, GE-Healthcare, Amersham™; ALEXA fluor^®^ 594-labeled anti-mouse antibody, Molecular Probes; both 1:1000, at RT for 60 min); DNA was then stained with 4′,6-diamidino-2-phenylindole (DAPI; QBiogene). A confocal fluorescence microscope (Zeiss Axioplan 2; 630-fold magnification) was used for analysis. At least 100 nuclei with γH2AX/53BP1 double positive foci were randomly selected and counted. Only intact nuclei were analyzed.

### Flow cytometry

Cells were stained with an EGFRvIII-specific antibody as described above (Immunofluorescence). Samples were analyzed using flow cytometry (FACScan Canto, BD Biosciences).

### Establishment of EGFRvIII+/− sublines

Parental DKMG and BS153 cell cultures expressing EGFRvIII heterogeneously were used to establish EGFRvIII+/− sublines. Cells cultures incubated in phosphate buffered saline containing 3 mM EDTA (PBS/EDTA, 15 min, 37°C) were detached by scraping, followed by an incubation with anti-EGFRvIII antibody (1 μg/ml) for 1 h at 4°C. Cells were then washed two times with PBS/EDTA and exposed to a secondary antibody (Alexa fluor^®^ 647, Molecular Probes) for 1 h at 4°C. After additional washing, cells were sorted according to either absent or maximal EGFRvIII expression using an ARIA III cytometer (BD Biosciences; FACS core facility of the University Medical Center Hamburg-Eppendorf). The separated EGFRvIII −/+ sublines were cultured in heat inactivated FCS. In both sublines, the level of EGFRvIII expression was found to be stable for at least 15–20 passages.

### Data evaluation

Unless otherwise indicated, experiments were repeated at least three times. The data are presented as mean values (±SEM). Prism software (GraphPad Prism 5, Firma) was used for analyzing and graphing the data. The unpaired student's *t*-test was performed for the statistical analysis. *P*-values were calculated using two-sided tests (*p < 0.05; **p < 0.01; ***p < 0.001).

## SUPPLEMENTARY FIGURES AND TABLES


