# Supplementary material for: EGFRvIII does not affect radiosensitivity with or without gefitinib treatment in glioblastoma cells
Source: Oncotarget. 2015 Sep 16;6(32):33867–77. doi: 10.18632/oncotarget.5293 (PMC4741808; doi:10.18632/oncotarget.5293)
Supplement: Supplementary file 1 [file oncotarget-06-33867-s001.pdf]

SUPPLEMENTARY FIGURES AND TABLES

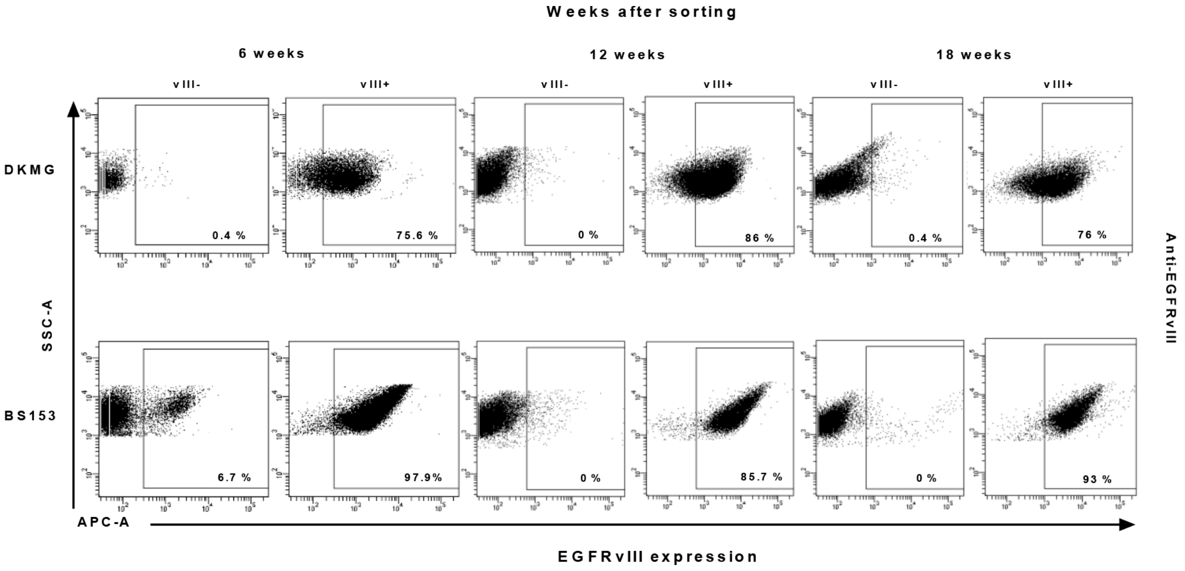

Supplementary Figure S1: EGFRvIII expression in EGFRvIII+ and EGFRvIII– sublines at different time points. EGFRvIII expression was quantified in DKMG and BS153 sub-lines by flow cytometry (dot plots). EGFRvIII was detected using an EGFRvIII-specific antibody (APC-A; SSC-A: side scatter). Cells were analzed at different time points: 6 weeks (6 passages), 12 weeks (13 passages) and 18 weeks (20 passages) after sorting.

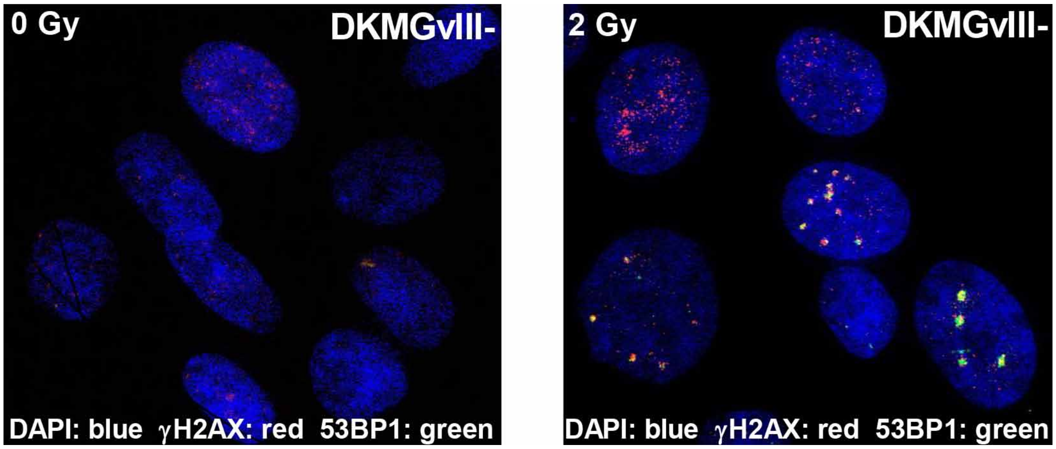

Supplementary Figure S2: Impact of EGFRvIII expression on DSB repair capacity. Residual DSB repair foci detected by γH2AX (red) and 53BP1 (green) immunofluorescent staining in DKMGvIII– cells 24 h after irradiation with 2 Gy (DNA staining with DAPI: blue). Quantification of residual γH2AX/53BP1 double positive foci in DKMGvIII−/+ and BS153vIII−/+ cells 24 h after irradiation with 2 Gy in Figure 4A.

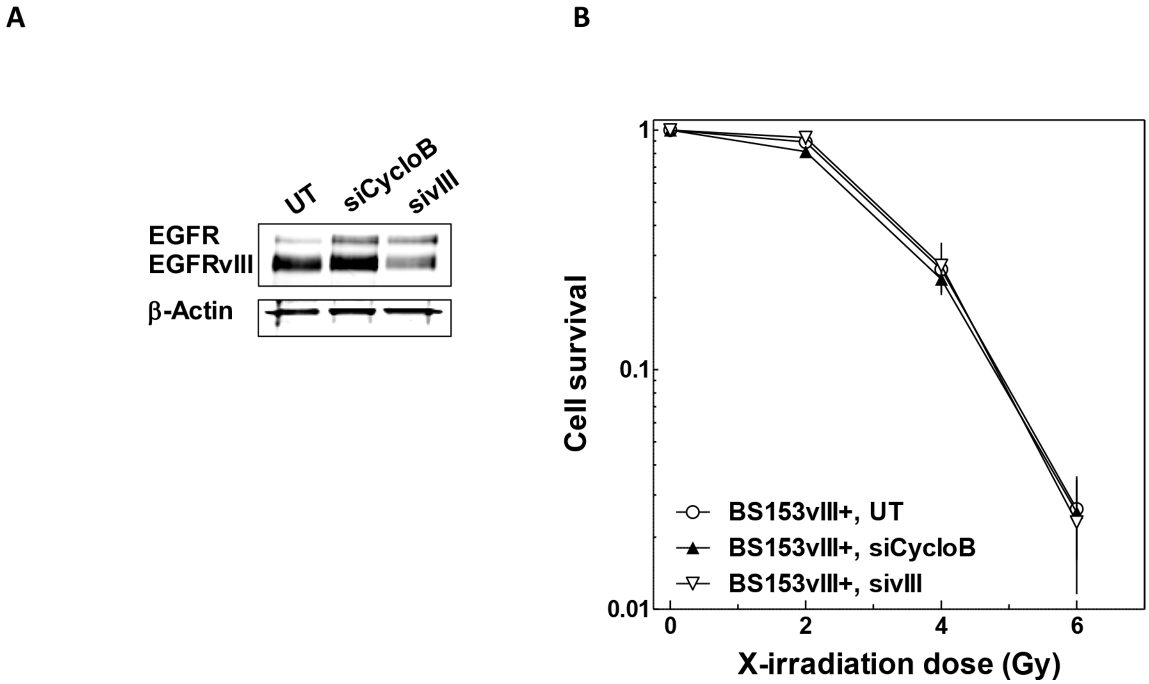

Supplementary Figure S3: Cell survival after EGFRvIII knock down. Cell survival after EGFRvIII specific knock down was determined by colony forming assay. 1×10^5 cells were seeded per 6-well-plate and incubated for 30 min to enable attachment of cells. Cells were then transfected with 30 nM EGFRvIII specific siRNA or 30 nM Cyclophilin B siRNA as a control using Hyperfect (Quiagen) according to manufacturer instructions. Knock down was performed for 48 h hours. **A.** To determine the efficiency of the EGFRvIII specific knock down the cells were harvested, counted and prepared for Western Blot analysis. **B.** For colony formation assay 400 cells were seeded per 6-well plate 24 h prior irradiation. The medium was replaced 24 h after treatment, followed by further incubation with AmnioMax C-100 Basal Medium (Life Technologies) containing 10% FCS and C-100 supplement (Life Technologies) to optimize colony formation. Colonies were allowed to grow for 3 weeks. Colonies were then fixed in 70% ethanol and stained with crystal violet; colonies of more than 50 cells were counted. The surviving fraction of irradiated cells was normalized to the plating efficiency of un-irradiated cells. One representative experiment is shown.

Supplementary Table S1: Genetic characterization of EGFRvIII−/+ sublines

Parameter	DKMG		BS153	
	vIII–	vIII+	vIII–	vIII+
**PTEN**	C136Y	C136Y	T167A	T167A
**p53**	WT	WT	R248Q	R248Q
**DNA content***	1	1	1.9	1.9
**Chromosomes****	43.6 (36–49)	43.6 (37–49)	81.3 (72–90)	80.7 (75–95)

*x-fold compared to normal lymphocytes
**average number of chromosomes (min/max)

Supplementary Table S2: Authentication of EGFRvIII−/+ sublines

Dye	Locus	Control theor.*	BS153vIII–	BS153vIIIs+	DKMGvIII–	DKMGvIII+
6-FAM	D8S1179	13	13	13	11 / 13	13
	D21S11	30	28 / 29	28 / 29	29 / 30 / 32.2	29 / 30
	D7S820	10 / 11	9 / 11	9 / 11	9 / 12	9 / 12
	CSF1PO	10 / 12	10 / 12	10 / 12	10	10
VIC	D3S1358	14 / 15	17	17	15 / 16 / 17	15 / 16
	TH01 11	8 / 9.3	6 / 9	6 / 9	9.3	9.3
	D13S317	11	12	12	8 / 11	8 / 11
	D16S539	11 /12	9	9	10 / 11 / 12	10 / 11
	D2S1338	19 / 23	17 /25	17 / 25	20 / 23 / 24 / 25	24 / 25
NED	D19S433	14 / 15	14 / 16.2	14 / 16.2	13 / 15.2	13 / 15.2
	vWA 12	17 / 18	15 / 18	15 / 18	17 / 18 / 19	18 / 19
	TPOX 2	8	11	11	8	8
	D18S51	15 / 19	12 / 17	12 / 17	13 / 17	13 / 17
PAT	Amel X,Y	X	X	X	X	X
	D5S818	11	11 / 13	11 / 13	9 / 11	9 / 11
	FGA 4	23 / 24	21 / 22	21 / 22	19 / 24	19 / 24

(AmpFlSTR® Itentifiler® PCR Amplification Kit
*GeneScan™ – 600 LIZ® Size Standard, Applied Biosystems)
